# Ethyl 5-((1*E*)-1-{(*E*)-2-[1-(4-eth­oxy­carbonyl-3-methyl-1,2-oxazol-5-yl)ethyl­idene]hydrazin-1-yl­idene}eth­yl)-3-methyl-1,2-oxazole-4-carboxyl­ate

**DOI:** 10.1107/S1600536811032004

**Published:** 2011-08-11

**Authors:** Abdullah M. Asiri, Abdulrahman O. Al-Youbi, Hassan M. Faidallah, Seik Weng Ng, Edward R. T. Tiekink

**Affiliations:** a Chemistry Department, Faculty of Science, King Abdulaziz University, PO Box 80203, Jeddah, Saudi Arabia; b The Center of Excellence for Advanced Materials Research, King Abdulaziz University, Jeddah, PO Box 80203, Saudi Arabia; c Department of Chemistry, University of Malaya, 50603 Kuala Lumpur, Malaysia

## Abstract

The complete mol­ecule of the title compound, C_18_H_22_N_4_O_6_, is generated by the application of a twofold axis of symmetry. Twists are evident in the mol­ecule, *i.e*. between each —C=N—N group and the adjacent oxazole ring [dihedral angle = 46.08 (12) °] and between the latter and attached ester group [excluding the terminal methyl group; dihedral angle = 24.4 (7) °]. In the crystal, C—H⋯O and π–π [3.5990 (11) Å] contacts connect mol­ecules into supra­molecular arrays in the *ac* plane. These stack along the *b* axis, being connected by weak π–π [3.3903 (11) Å] inter­actions.

## Related literature

For background to the biological activity of hydrazone compounds, see: Faid-Allah *et al.* (2011 ▶).
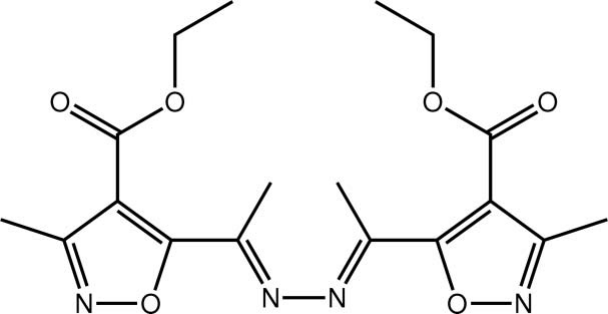


## Experimental

### 

#### Crystal data

C_18_H_22_N_4_O_6_
*M* *_r_* = 390.40
Monoclinic, 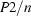

*a* = 9.4509 (5) Å
*b* = 8.5456 (4) Å
*c* = 11.9859 (5) Å
β = 104.107 (5)°
*V* = 938.83 (8) Å^3^
*Z* = 2
Mo *K*α radiation
μ = 0.11 mm^−1^
*T* = 100 K
0.25 × 0.25 × 0.05 mm

#### Data collection

Agilent SuperNova Dual diffractometer with Atlas detector
Absorption correction: multi-scan (*CrysAlis PRO*; Agilent, 2010 ▶) *T*_min_ = 0.889, *T*_max_ = 1.000
4223 measured reflections
2095 independent reflections
1639 reflections with *I* > 2σ(*I*)
*R*_int_ = 0.023

#### Refinement

*R*[*F* ^2^ > 2σ(*F* ^2^)] = 0.049
*wR*(*F* ^2^) = 0.159
*S* = 0.87
2095 reflections
129 parameters
H-atom parameters constrained
Δρ_max_ = 0.37 e Å^−3^
Δρ_min_ = −0.32 e Å^−3^

### 

Data collection: *CrysAlis PRO* (Agilent, 2010 ▶); cell refinement: *CrysAlis PRO*; data reduction: *CrysAlis PRO*; program(s) used to solve structure: *SHELXS97* (Sheldrick, 2008 ▶); program(s) used to refine structure: *SHELXL97* (Sheldrick, 2008 ▶); molecular graphics: *ORTEP-3* (Farrugia, 1997 ▶) and *DIAMOND* (Brandenburg, 2006 ▶); software used to prepare material for publication: *publCIF* (Westrip, 2010 ▶).

## Supplementary Material

Crystal structure: contains datablock(s) global, I. DOI: 10.1107/S1600536811032004/hg5078sup1.cif

Structure factors: contains datablock(s) I. DOI: 10.1107/S1600536811032004/hg5078Isup2.hkl

Supplementary material file. DOI: 10.1107/S1600536811032004/hg5078Isup3.cml

Additional supplementary materials:  crystallographic information; 3D view; checkCIF report

## Figures and Tables

**Table 1:** Hydrogen-bond geometry (Å, °)

*D*—H⋯*A*	*D*—H	H⋯*A*	*D*⋯*A*	*D*—H⋯*A*
C9—H9c⋯O2^i^	0.98	2.46	3.356 (3)	152

Symmetry code: (i) 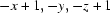.

**Fig. 1.**
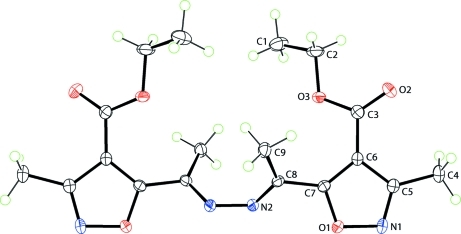
The molecular structure of (I) showing the atom-labelling scheme and displacement ellipsoids at the 50% probability level. The molecule has crystallographic 2-fold symmetry and unlabelled atoms are generated by the symmetry operation 0.5 - x, y, 1.5 - z.

**Fig. 2.**
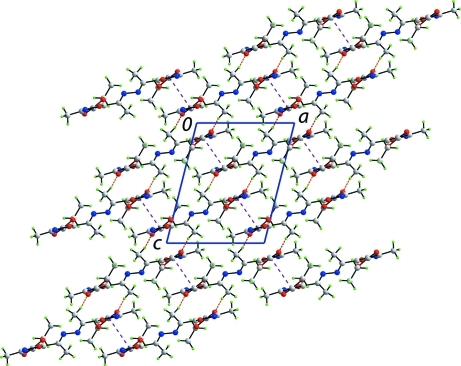
Supramolecular array in the ac plane in (I) mediated by C—H···O and π···π interactions shown as orange and purple dashed lines, respectively.

**Fig. 3.**
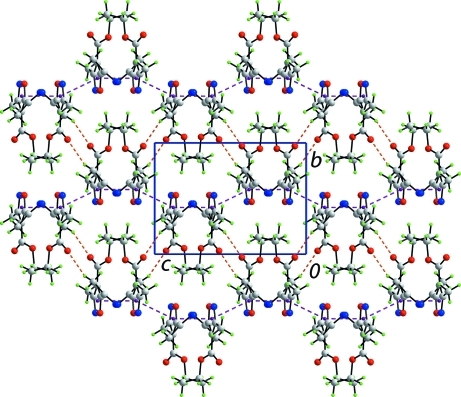
A view in projection down the a axis of the unit-cell contents of (I). The C—H···O and π···π interactions are shown as orange and purple dashed lines, respectively.

## supplementary crystallographic information

### Comment

The study of the title compound (I) was motivated by the recent report of the significant anti-bacterial and anti-fungal activity exhibited hydrazone compounds (Faid-Allah *et al.*, 2011).

The full molecule of (I) is generated by the application of a 2-fold axis of symmetry. The configuration about the imine bond [1.280 (3) Å] is *E*. There are significant twists throughout the molecule. Firstly, the oxazole ring [r.m.s. deviation = 0.007 Å] is twisted away from the plane of the central —C═N—N═C— group as seen in the value of the O1—C7—C8—N2 torsion angle of -43.3 (2)°. Further, the ester group lies out of the plane through the oxazole ring with the O2—C3—C6—C7 torsion angle being 160.5 (2) °. The oxazole-O atoms as well as the ester-ethyl groups are orientated towards the 2-fold axis while the carbonyl-O atoms are directed away from the axis. The terminal methyl group of the ester lies out of the plane of the remaining non-H atoms [the C3—O3—C2—C1 torsion angle = 159.33 (19) °].

Both C—H···O, Table 1, and π···π interactions feature in the crystal packing. The C—H···O and π···π contacts between oxazole rings [3.5990 (11) Å for symmetry operation 3/2 - *x*, *y*, 1.5 - *z*] combine to link molecules into supramolecular arrays in the *ac* plane, Fig. 2. These partially interdigitate with centrosymmetrically related layers along the *b* axis allowing for the formation of additional π···π interactions [3.3903 (11) Å for symmetry operation 1 - *x*, 1 - *y*, 1 - *z*], Fig. 3.

### Experimental

Ethyl 5-acetyl-2-methylthiazole-4-carboxylate (10 mmol) in C_2_H_5_OH (25 ml) was refluxed with hydrazine hydrate (12 mmol) for 1 h. The hydrazone which separated after concentration of the reaction mixture was filtered off, washed with C_2_H_5_OH, and recrystallized from C_2_H_5_OH; *M*.pt. 448 K.

### Refinement

Carbon-bound H-atoms were placed in calculated positions [C—H 0.98 to 0.99 Å, *U*_iso_(H) 1.2 to 1.5*U*_eq_(C)] and were included in the refinement in the riding model approximation.

### Crystal data

**Table:**

C_18_H_22_N_4_O_6_	*F*(000) = 412
*M**_r_* = 390.40	*D*_x_ = 1.381 Mg m^−^^3^
Monoclinic, *P*2/*n*	Mo *K*α radiation, λ = 0.71073 Å
Hall symbol: -P 2yac	Cell parameters from 1742 reflections
*a* = 9.4509 (5) Å	θ = 2.4–29.3°
*b* = 8.5456 (4) Å	µ = 0.11 mm^−^^1^
*c* = 11.9859 (5) Å	*T* = 100 K
β = 104.107 (5)°	Plate, colourless
*V* = 938.83 (8) Å^3^	0.25 × 0.25 × 0.05 mm
*Z* = 2	

### Data collection

**Table:**

Agilent SuperNova Dual diffractometer with Atlas detector	2095 independent reflections
Radiation source: SuperNova (Mo) X-ray Source	1639 reflections with *I* > 2σ(*I*)
mirror	*R*_int_ = 0.023
Detector resolution: 10.4041 pixels mm^-1^	θ_max_ = 27.5°, θ_min_ = 2.4°
ω scans	*h* = −11→11
Absorption correction: multi-scan (*CrysAlis PRO*; Agilent, 2010)	*k* = −11→10
*T*_min_ = 0.889, *T*_max_ = 1.000	*l* = −14→15
4223 measured reflections	

### Refinement

**Table:**

Refinement on *F*^2^	Primary atom site location: structure-invariant direct methods
Least-squares matrix: full	Secondary atom site location: difference Fourier map
*R*[*F*^2^ > 2σ(*F*^2^)] = 0.049	Hydrogen site location: inferred from neighbouring sites
*wR*(*F*^2^) = 0.159	H-atom parameters constrained
*S* = 0.87	*w* = 1/[σ^2^(*F*_o_^2^) + (0.0921*P*)^2^ + 1.4075*P*] where *P* = (*F*_o_^2^ + 2*F*_c_^2^)/3
2095 reflections	(Δ/σ)_max_ < 0.001
129 parameters	Δρ_max_ = 0.37 e Å^−^^3^
0 restraints	Δρ_min_ = −0.32 e Å^−^^3^

### Special details

**Table:**

Geometry. All s.u.'s (except the s.u. in the dihedral angle between two l.s. planes) are estimated using the full covariance matrix. The cell s.u.'s are taken into account individually in the estimation of s.u.'s in distances, angles and torsion angles; correlations between s.u.'s in cell parameters are only used when they are defined by crystal symmetry. An approximate (isotropic) treatment of cell s.u.'s is used for estimating s.u.'s involving l.s. planes.
Refinement. Refinement of *F*^2^ against ALL reflections. The weighted *R*-factor *wR* and goodness of fit *S* are based on *F*^2^, conventional *R*-factors *R* are based on *F*, with *F* set to zero for negative *F*^2^. The threshold expression of *F*^2^ > 2σ(*F*^2^) is used only for calculating *R*-factors(gt) *etc*. and is not relevant to the choice of reflections for refinement. *R*-factors based on *F*^2^ are statistically about twice as large as those based on *F*, and *R*- factors based on ALL data will be even larger.

### Fractional atomic coordinates and isotropic or equivalent isotropic displacement parameters (Å^2^)

**Table:**

	*x*	*y*	*z*	*U*_iso_*/*U*_eq_	
O1	0.54005 (15)	0.52943 (17)	0.63908 (12)	0.0176 (3)	
O2	0.67094 (17)	0.02949 (18)	0.57529 (14)	0.0248 (4)	
O3	0.51960 (17)	0.05289 (17)	0.69403 (12)	0.0202 (4)	
N1	0.67630 (19)	0.5276 (2)	0.60982 (14)	0.0186 (4)	
N2	0.32058 (18)	0.45172 (19)	0.74369 (14)	0.0162 (4)	
C1	0.4715 (3)	−0.1526 (3)	0.8137 (2)	0.0309 (6)	
H1A	0.4662	−0.2662	0.8231	0.046*	
H1B	0.3756	−0.1062	0.8101	0.046*	
H1C	0.5434	−0.1085	0.8792	0.046*	
C2	0.5158 (3)	−0.1174 (3)	0.7054 (2)	0.0271 (5)	
H2A	0.6133	−0.1623	0.7086	0.033*	
H2B	0.4450	−0.1632	0.6387	0.033*	
C3	0.5997 (2)	0.1086 (3)	0.62488 (16)	0.0172 (4)	
C4	0.8420 (2)	0.3360 (3)	0.56290 (18)	0.0226 (5)	
H4A	0.8956	0.4309	0.5526	0.034*	
H4B	0.8161	0.2777	0.4904	0.034*	
H4C	0.9033	0.2705	0.6227	0.034*	
C5	0.7064 (2)	0.3801 (2)	0.59804 (16)	0.0161 (4)	
C6	0.5953 (2)	0.2805 (2)	0.62067 (15)	0.0150 (4)	
C7	0.4951 (2)	0.3805 (2)	0.64479 (16)	0.0148 (4)	
C8	0.3485 (2)	0.3616 (2)	0.66646 (16)	0.0153 (4)	
C9	0.2435 (2)	0.2483 (3)	0.59511 (18)	0.0201 (5)	
H9A	0.1556	0.3042	0.5542	0.030*	
H9B	0.2170	0.1685	0.6451	0.030*	
H9C	0.2892	0.1980	0.5393	0.030*	

### Atomic displacement parameters (Å^2^)

**Table:**

	*U*^11^	*U*^22^	*U*^33^	*U*^12^	*U*^13^	*U*^23^
O1	0.0160 (7)	0.0156 (8)	0.0229 (7)	−0.0016 (5)	0.0082 (6)	0.0009 (6)
O2	0.0251 (9)	0.0214 (8)	0.0312 (8)	0.0041 (6)	0.0134 (7)	−0.0046 (6)
O3	0.0276 (8)	0.0123 (7)	0.0238 (8)	0.0014 (6)	0.0124 (6)	0.0017 (6)
N1	0.0141 (8)	0.0240 (10)	0.0194 (8)	−0.0022 (7)	0.0074 (7)	0.0010 (7)
N2	0.0142 (9)	0.0152 (9)	0.0200 (8)	0.0015 (6)	0.0059 (7)	0.0020 (7)
C1	0.0438 (15)	0.0198 (12)	0.0295 (12)	−0.0039 (10)	0.0099 (11)	0.0041 (9)
C2	0.0397 (14)	0.0120 (11)	0.0323 (12)	0.0001 (9)	0.0138 (10)	0.0017 (9)
C3	0.0146 (10)	0.0192 (11)	0.0168 (9)	0.0006 (8)	0.0017 (8)	−0.0006 (8)
C4	0.0159 (10)	0.0304 (12)	0.0235 (10)	0.0016 (9)	0.0086 (8)	0.0012 (9)
C5	0.0153 (9)	0.0201 (10)	0.0126 (9)	0.0000 (8)	0.0026 (7)	0.0014 (7)
C6	0.0133 (9)	0.0182 (10)	0.0138 (9)	0.0004 (7)	0.0037 (7)	0.0010 (7)
C7	0.0162 (10)	0.0143 (10)	0.0138 (9)	−0.0010 (8)	0.0034 (7)	0.0014 (7)
C8	0.0155 (10)	0.0129 (9)	0.0181 (9)	−0.0004 (7)	0.0049 (7)	0.0030 (7)
C9	0.0166 (10)	0.0226 (11)	0.0220 (10)	−0.0030 (8)	0.0065 (8)	−0.0034 (8)

### Geometric parameters (Å, °)

**Table:**

O1—C7	1.349 (2)	C2—H2B	0.9900
O1—N1	1.415 (2)	C3—C6	1.470 (3)
O2—C3	1.207 (2)	C4—C5	1.492 (3)
O3—C3	1.339 (2)	C4—H4A	0.9800
O3—C2	1.462 (3)	C4—H4B	0.9800
N1—C5	1.307 (3)	C4—H4C	0.9800
N2—C8	1.280 (3)	C5—C6	1.428 (3)
N2—N2^i^	1.379 (3)	C6—C7	1.358 (3)
C1—C2	1.489 (3)	C7—C8	1.479 (3)
C1—H1A	0.9800	C8—C9	1.496 (3)
C1—H1B	0.9800	C9—H9A	0.9800
C1—H1C	0.9800	C9—H9B	0.9800
C2—H2A	0.9900	C9—H9C	0.9800
			
C7—O1—N1	108.63 (14)	C5—C4—H4C	109.5
C3—O3—C2	116.20 (16)	H4A—C4—H4C	109.5
C5—N1—O1	105.79 (16)	H4B—C4—H4C	109.5
C8—N2—N2^i^	117.04 (17)	N1—C5—C6	111.43 (18)
C2—C1—H1A	109.5	N1—C5—C4	119.89 (19)
C2—C1—H1B	109.5	C6—C5—C4	128.67 (19)
H1A—C1—H1B	109.5	C7—C6—C5	104.37 (18)
C2—C1—H1C	109.5	C7—C6—C3	129.58 (18)
H1A—C1—H1C	109.5	C5—C6—C3	125.88 (18)
H1B—C1—H1C	109.5	O1—C7—C6	109.77 (17)
O3—C2—C1	107.50 (18)	O1—C7—C8	115.58 (17)
O3—C2—H2A	110.2	C6—C7—C8	134.47 (19)
C1—C2—H2A	110.2	N2—C8—C7	115.36 (17)
O3—C2—H2B	110.2	N2—C8—C9	125.31 (18)
C1—C2—H2B	110.2	C7—C8—C9	119.26 (17)
H2A—C2—H2B	108.5	C8—C9—H9A	109.5
O2—C3—O3	125.0 (2)	C8—C9—H9B	109.5
O2—C3—C6	123.86 (19)	H9A—C9—H9B	109.5
O3—C3—C6	111.13 (17)	C8—C9—H9C	109.5
C5—C4—H4A	109.5	H9A—C9—H9C	109.5
C5—C4—H4B	109.5	H9B—C9—H9C	109.5
H4A—C4—H4B	109.5		
			
C7—O1—N1—C5	0.7 (2)	O3—C3—C6—C5	152.60 (18)
C3—O3—C2—C1	159.33 (19)	N1—O1—C7—C6	0.0 (2)
C2—O3—C3—O2	−2.3 (3)	N1—O1—C7—C8	−175.79 (15)
C2—O3—C3—C6	−179.88 (17)	C5—C6—C7—O1	−0.6 (2)
O1—N1—C5—C6	−1.1 (2)	C3—C6—C7—O1	174.79 (18)
O1—N1—C5—C4	177.90 (16)	C5—C6—C7—C8	174.0 (2)
N1—C5—C6—C7	1.1 (2)	C3—C6—C7—C8	−10.5 (4)
C4—C5—C6—C7	−177.79 (19)	N2^i^—N2—C8—C7	172.11 (15)
N1—C5—C6—C3	−174.53 (18)	N2^i^—N2—C8—C9	−4.7 (3)
C4—C5—C6—C3	6.6 (3)	O1—C7—C8—N2	−43.3 (2)
O2—C3—C6—C7	160.5 (2)	C6—C7—C8—N2	142.2 (2)
O3—C3—C6—C7	−21.9 (3)	O1—C7—C8—C9	133.71 (19)
O2—C3—C6—C5	−25.0 (3)	C6—C7—C8—C9	−40.7 (3)

Symmetry codes: (i) −*x*+1/2, *y*, −*z*+3/2.

### Hydrogen-bond geometry (Å, °)

**Table:**

*D*—H···*A*	*D*—H	H···*A*	*D*···*A*	*D*—H···*A*
C9—H9c···O2^ii^	0.98	2.46	3.356 (3)	152

Symmetry codes: (ii) −*x*+1, −*y*, −*z*+1.
